# Population-Based Laboratory Surveillance of Imported Malaria in Metropolitan Calgary, 2000–2011

**DOI:** 10.1371/journal.pone.0060751

**Published:** 2013-04-15

**Authors:** Clara S. Lee, Daniel B. Gregson, Deirdre Church, Kevin B. Laupland, Rose Eckhardt, Terry Ross, Wilson Chan, Dylan R. Pillai

**Affiliations:** 1 Calgary Laboratory Services, Calgary, Alberta, Canada; 2 Department of Pathology and Laboratory Medicine and Medicine, The University of Calgary, Calgary, Alberta, Canada; 3 Department of Microbiology, Immunology, and Infectious Diseases, The University of Calgary, Calgary, Alberta, Canada; 4 Keenan Research Centre, Li Ka Shing Knowledge Institute, St Michael's Hospital, Toronto, Ontario, Canada; Johns Hopkins Bloomberg School of Public Health, United States of America

## Abstract

Increased travel leads to a heightened risk of imported infectious diseases. Patterns of immigration to countries like Canada have changed such that countries of malaria endemicity are frequented in larger numbers. In keeping with the changes in travel patterns and immigration, the major metropolitan city of Calgary has seen a dramatic rise in malaria incidence over the last decade. Fuelling this rise in Calgary has been the apparent complacence with prophylaxis in individuals visiting friends and relatives and potentially inadequate public health intervention in areas of the city with increased immigration and lower socioeconomic status.

## Introduction

Malaria is a devastating global health concern as its high morbidity and mortality pose threats to many populations around the world. In the mid 20^th^ century, in an attempt to suppress the spreading of malaria, the World Health Organization (WHO) established the Global Malaria Eradication campaign [1]. However, since the development of chloroquine resistance in *Plasmodium falciparum* strains and the development of DDT resistance in *Anopheles* mosquitoes, malaria has returned into many areas that were previously controlled [1]. Today, it has been estimated that there are 216 million malaria related cases of infections, and 655,000 deaths a year [2]. The situation is further worsened with the finding that 3.3 billion people are exposed and at risk of contracting malaria [2].

The geographical distribution pattern of malaria is highly confined, but it is not limited to tropics and sub-tropics. Annually, 80 to 90 million people from malaria non-endemic countries visit developing countries with high malaria transmission rates [3]. With the continuous expansion and diversification of intercontinental travel, occurrences of malaria within the non-endemic countries is becoming more evident [4], [5]. In Canada, approximately 500 malaria cases are reported each year although no formal surveillance system is in place [6]. Analogous to the European countries and United States, disproportionately large amounts of malaria in Canada are imported from overseas [7], [8]. Canada's reported malaria cases are densely concentrated within urban centres of Ontario, British Columbia, and Quebec [6]. Of note, the three provinces with the highest malaria rates also experience the highest immigration rates [9]. Past studies have suggested that immigrants originating from malaria endemic countries returning to their countries to visit their friends and relatives (VFR) have higher risk of acquiring malaria in comparison to other travellers [10]–[12]. The occurrence is often related to the VFR traveller's tendency to underestimate the risk of contracting malaria, and thereby not adhering to or seeking advice on prevention measures [13].

Calgary is becoming one the fastest growing metropolitan cities in Canada, exhibiting steady growth in immigrant [14]. In 2010, nearly 30% (1,091,000) of Calgary's inhabitants were comprised of immigrants [15]. Along with the emergence of people from different ethnic backgrounds, it has been observed anecdotally that the number of imported malaria cases are increasing in Calgary. Calgary's consolidated laboratory testing for malaria enables population-based analysis of trends in imported malaria [16]. Therefore, the main objective of this study was to retrospectively analyze the epidemiological characteristics and trends of imported malaria among returning travellers and incoming immigrants to Calgary, Canada, between the years 2000 and 2011. This epidemiological profile will allow for local public health risk assessment of malaria, and thereby the enhancement of prevention, diagnosis, and treatment.

## Materials and Methods

### Study Population and Ethics

Ethics approval to conduct this study was obtained from the Conjoint Health Research Ethics Board at the University of Calgary (CHREB Ethics ID 24835). Patient consent was waived per CHREB as this was a retrospective study with aggregate data containing no patient identifiers. This study included patients who tested positive for malaria in Calgary between January 2000 and December 2011. Every suspected malaria case within Calgary was exclusively reported to Calgary Laboratory Services (CLS), a centralized regional medical diagnostic laboratory, for clinical testing [16]. In 2011, Calgary's total population was 1,090,936 [17].

### Laboratory Diagnosis

Laboratory's diagnostic workflow involved rapid diagnostic test (BinaxNOW, Alere, Ottawa, ON) as a screen and microscopy using thick and thin Giemsa-stained peripheral blood smears for confirmation as previously described [16]. Specimens were also forwarded to Toronto General Hospital or the Provincial Laboratory in Edmonton for real-time polymerase chain reaction (PCR) confirmation [18], [19]. Laboratory data were used for parasite identification and quantification. When calculating the geometric mean parasitaemia (GMP), the following considerations were made: 1) samples that only contained gametocytes or with missing information were not included in the calculation, 2) parasite load values were adjusted to 0.1% for any samples where a parsitemia was not available. Discordant results are described in the next section.

### Reporting a Case

When reporting a case, the following considerations were made: 1) Relapsing infections that were less than 2 months apart were counted as a single case, 2) infections associated with more than one type of *Plasmodium* species were counted as a single case and were classified as a mixed infection, 3) in case of discordant results between the types of *Plasmodium* species that patients were infected with, real-time PCR assay results were always prioritized over results that were identified by other detection methods (i.e. microscopy and rapid diagnostic test), 4) discordant results that could not be distinguished by the real-time PCR (i.e. invalid/unavailable PCR results) were counted as a single case, and were classified as *Plasmodium* spp.

### Data Collection

A standardized clinical history form was requested for all patients with samples submitted for malaria testing from 2007–2011. Demographic variables reviewed in this study were: gender, age and location of residence. Physicians were required to complete CLS malaria history forms upon requesting an order for specimen malaria testing. For this study, Malaria case history forms (n = 4832) were manually entered into a Microsoft access database. containing information on patient's travel and clinical history, which was further divided into different criteria including the following: pre-travel advice (yes or no), reason for travel (tourism, business, new immigrant, VFR, or visitor to Canada), countries with malaria visited, types of prophylaxis taken, and treatment (yes or no, and if yes list). Each section showed different completion rate, 100%, 81.7%, 93.7%, 100% (82.09% of the people who answered yes listed the name of the treatment), respectively. N/A refers to the sections with insufficient information.

When multiple history forms were completed by a single patient regarding their travel and clinical history, the original form was used and complemented with additional information made available from subsequent forms. Patients' postal codes were collected in order to determine the incidence rate of malaria within different city wards for year 2011. Calgary is divided into 14 different wards, and the incidence rate for the each ward is reported as the number of cases per 100,000 people.

### Data Processing and Statistical Analysis

The database was created using Microsoft Access in order to manage and update the information obtained from the patients' malaria history forms. Data held in Microsoft Access was then exported to Microsoft Excel for the facilitated data analysis. The ANOVA test was used for comparing the means of more than two groups. A p-value≤0.05 was used as the cut-off value for statistical significance between the means. The malaria incidence rate was determined based on the census data provided by the City of the Calgary and Alberta Health Services (AHS) [17]. Calgary is divided into 14 different wards by the City of the Calgary. The social districts divisions set by AHS differ slightly based on census data from the City of the Calgary ward division. Socioeconomic data corresponding to each of the social districts from 2006 was used. A choropleth map was created that displays the incidence rate of malaria cases for each ward. All mapping was completed using ArcMap 10.1 (ESRI, Redlands). Geographic boundary files of Calgary wards were obtained from the University of Calgary Library.

## Results

### Epidemiologic Analysis of Malaria Cases

**Figure 1:**
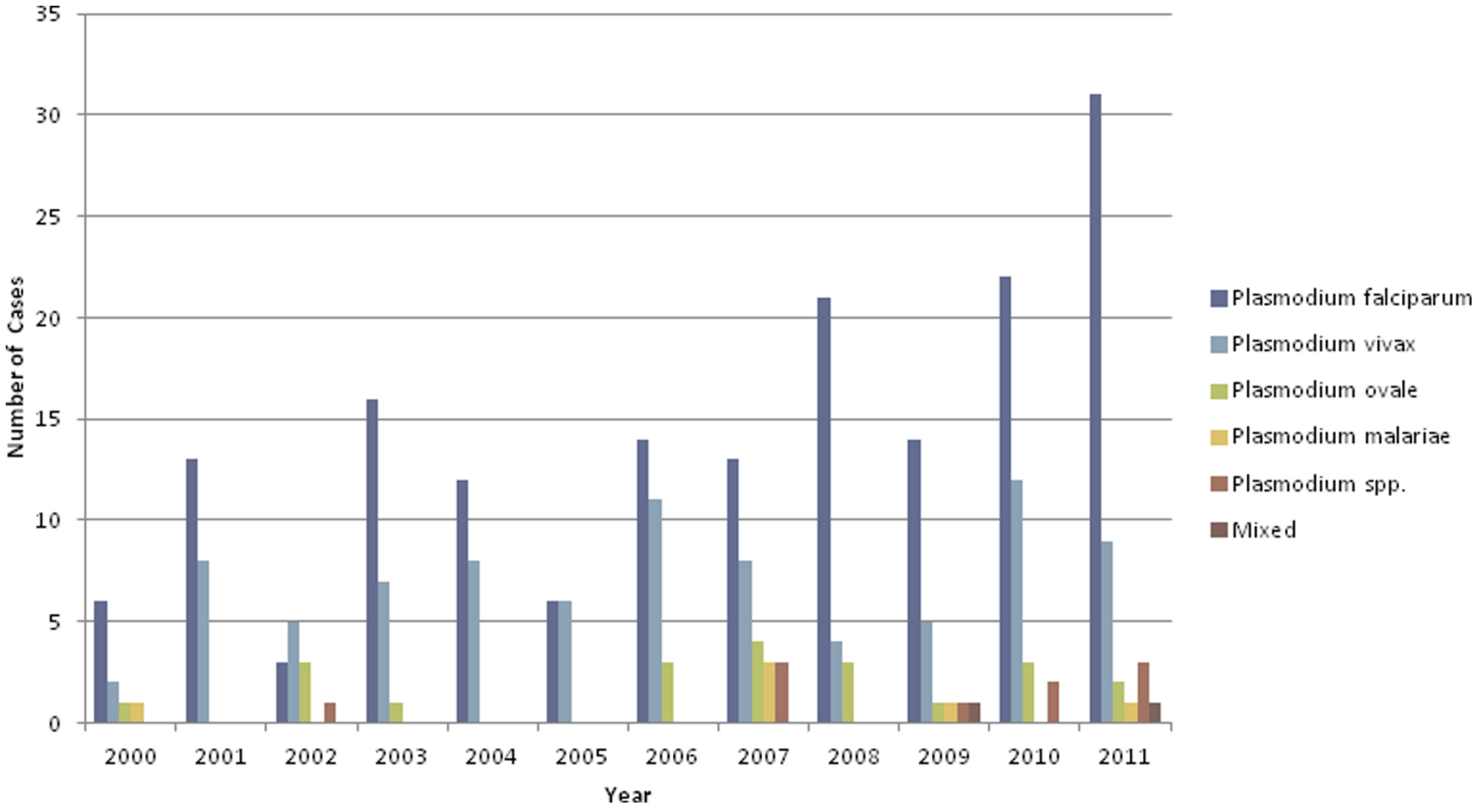
Number of Annual Malaria Cases by *Plasmodium* Species in Calgary, 2000–2011.

Between 2000 and 2011, there were 295 laboratory confirmed malaria cases (Figure 1). On average, 24.60±3.19 malaria cases were reported each year. During the 12-year period, there was an increased trend in the total number of annual cases and in the total number of *P. falciparum* infections (p = 0.05375). *P. falciparum* and *P. vivax* infections represented greater than 85% of the reported cases. Of the 295 cases, there were 171(58.0%) *P. falciparum* infections and 85 (28.8%) *P. vivax* infections. The remainder of the cases consisted of *P. ovale* (21, 7.1%), *P. malariae* (6, 2.0%), *Plasmodia* spp. (10, 3.4%) where speciation was not possible, and mixed infection (2, 0.7%). Patients classified as having a mixed infection were simultaneously infected with *P. falciparum* and *P. ovale*.

**Figure 2:**
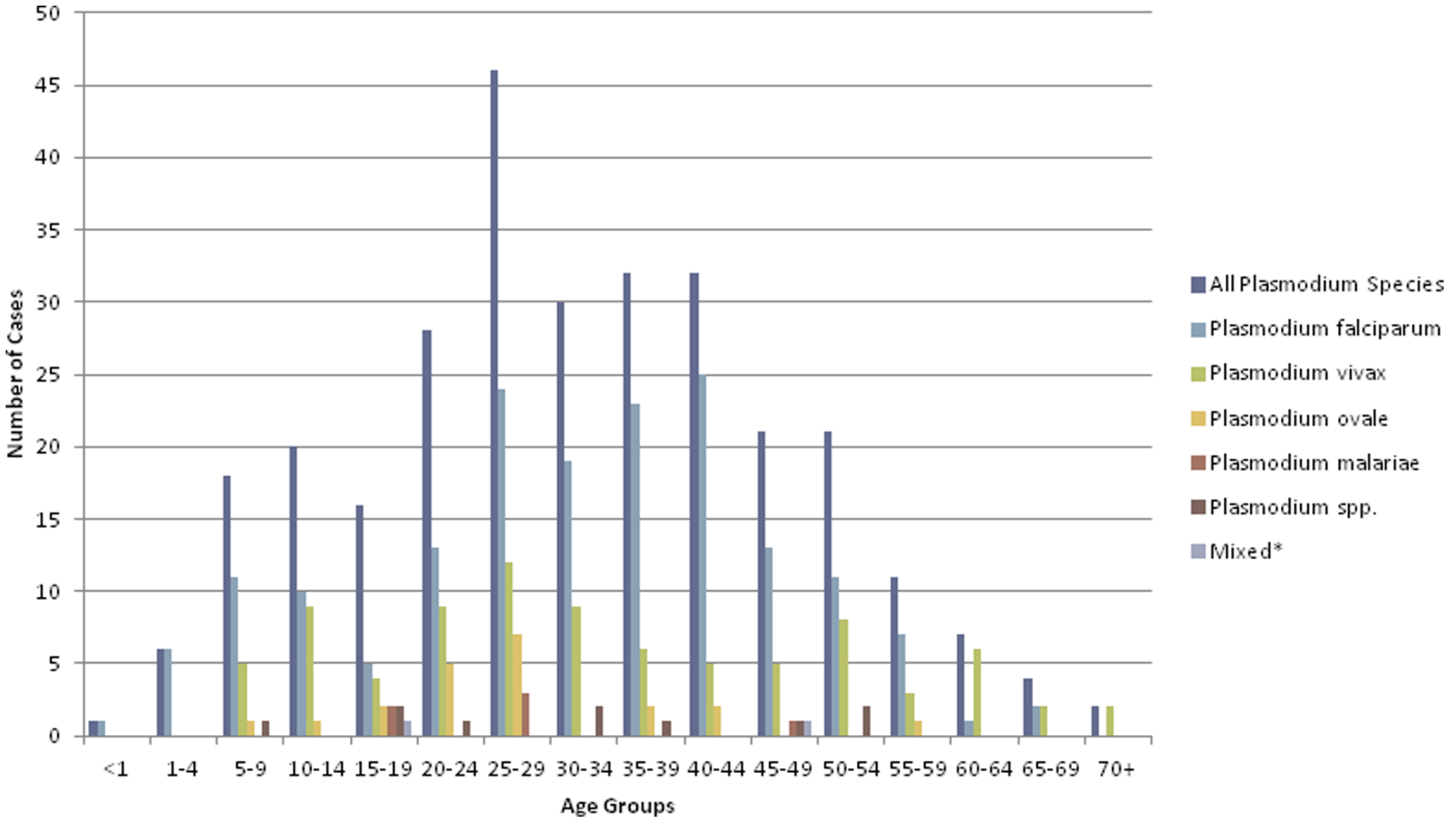
Number of Malaria Cases by Age group and *Plasmodium* Species in Calgary, 2000–2011.

The occurrence of malaria was significantly more frequent in males than in females (males, 16.83±2.64; females, 7.75±0.84 ; p<0.05) (Figure 2). *P.vivax* infections were predominant in males (p = 0.01), but for the other etiologic agents, the gender difference in the disease prevalence was insignificant. For all *Plasmodium* species, the mean age of onset of malaria was 32.03±1.20 years, with a wide age range of 0.8–87 years. One hundred and twenty four (73.8%) cases were observed in individuals between the age of 20 and 59, with the highest proportion in the 25–29 (15.5%) age group. Thirty seven cases (22.0%) were observed in infants and children <1–19, and 54.1% of the patients in this age group were infected with *P. falciparum*. Seven cases (4.2%) were observed in elderly individuals between the ages of 60–70+, and 71.4% of the patients within this age group were infected with *P. vivax*.

A minority of patients (23.8%) had received advice from a clinic/physician prior to their travel, but the percentage was slightly lower in infants and children (20.0%). Sixty percent of the individuals who had received pre-travel advice took prophylaxis, but the percentage of prophylaxis usage was reduced to 13.5% among the individuals who had not received pre-travel advice. The most common reason for travel was VFR (44%), followed by immigration to Canada (22.2%), tourism (11.1%), business (7.90%), and visiting Canada (1.60%) (Table 1). The mean age of the VFR travellers were 32±1.87 years, and 71.4% of the cases were males. The top three destinations for the VFR travellers were Sudan (25.0%), India (16.1%), and Nigeria (16.1%), and the greatest number of cases occurred in August. Tourists were most likely to take prophylaxis (42.9%), whereas prophylaxis was least likely to be taken by new immigrants (14.3%).

**Table 1:** Number and Percentage of Malaria cases by Reason for Travel in Calgary, 2007–2011.

Reason for Travel	Total Number of Cases (%)
Tourism	14 (11.1)
Business	10 (7.9)
New Immigrants	28 (22.2)
Visiting Friends and Relatives (VFR)	56 (44.4)
Visitor to Canada	2 (1.6)
N/A	23 (18.3)

**Figure 3:**
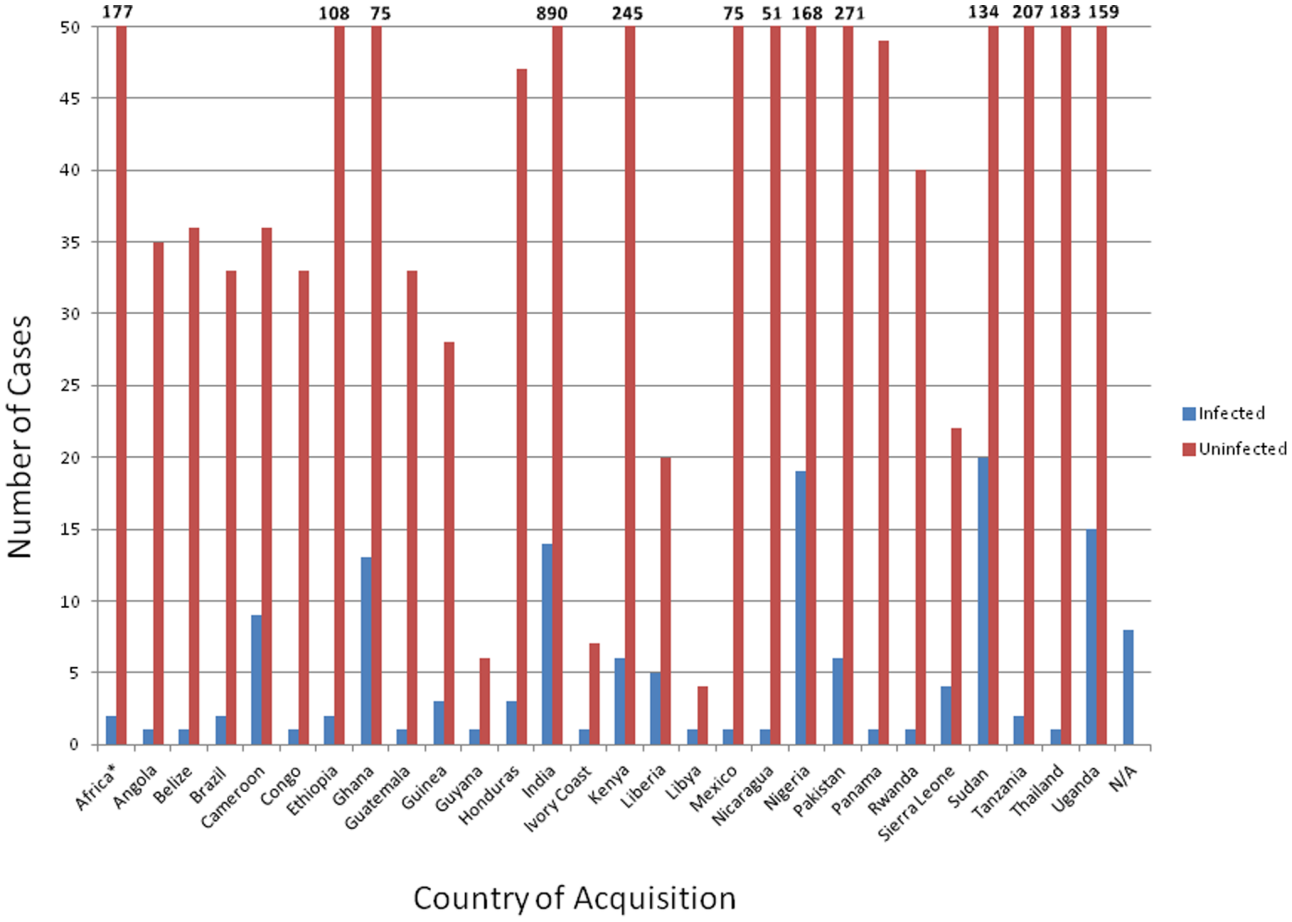
Number of Malaria Cases by Country of Acquisition in Calgary, 2007–2011.

All the reported malaria cases in Calgary were acquired outside Canada (Figure 3). During the five years for which we have travel data (2007–2011), 27 different countries (Africa, n = 17; Asia, n = 3; Americas, n = 6) were visited by the patients (Table 2). A majority of the etiologic agents were encountered in Africa. 80.0% of *P. falciparum*, 75.0% of *P. ovale*, 100.0% of *P. malariae*, and 100.0% of mixed infections were contracted in Africa. By contrast, the highest proportions of *P. vivax* (55.6%) and *Plasmodia* spp. (50.0%) infections were acquired in Asia. *P. vivax* was the only species contracted in the Americas (18.5% of all *P. vivax*). Overall, the highest numbers of cases originated from Sudan (n = 20), followed by Nigeria (n = 19), Uganda (n = 15), India (n = 14), Ghana (n = 13), and Cameroon (n = 9).

**Table 2:** Number and Percentage of Malaria Cases by Continent of Travel and *Plasmodium* Species in Calgary, 2007–2011.

	*Plasmodium falciparum*	*Plasmodium vivax*	*Plasmodium ovale*	*Plasmodium malariae*	*Plasmodium* spp.	Mixed	Total Infected	Total Uninfected Patients
Africa	74 (92.5)	2 (7.4)	7 (87.5)	4 (100)	2 (33.3)	2 (100)	91 (72.2)	1643 (33.7)
Asia	2 (2.5)	15 (55.6)	1 (12.5)	0 (0)	3 (50)	0 (0)	21 (16.7)	2230 (45.7)
Americas	0 (0)	5 (18.5)	0 (0)	0 (0)	0 (0)	0 (0)	5 (4.0)	1004 (20.6)
More than one continent with malaria visited	0 (0)	1 (3.7)	0(0)	0 (0)	0 (0)	0 (0)	1 (0.8)	-
N/A	4 (5.0)	3 (14.8)	0 (0)	0 (0)	1 (16.7)	0 (0)	8 (6.3)	-
Total	80	26	8	4	6	2	126 (100)	4877 (100)

Patients most commonly took doxycycline (n = 7) prophylaxis, followed by chloroquine (n = 5) (Table 3). Other prophylaxis (9.5%), which included Quinine (n = 2), artemether-lumefantrine (n = 1), Lumefantrine (n = 1), Proguanil (n = 1), Sulfadoxine-Pyrimethamine (n = 1), and six cases indicated that they took prophylaxis but did not know the name of the drug. Combination prophylaxis (4.5%) included chloroquine plus doxycycline, doxycycline plus quinine, or doxycycline plus quinine sulphate.

**Table 3:** Number and Percentage of Different Prophylactic Drug Used by Returning Travellers to Calgary, 2007–2011.

Types of Prophylaxis	Number of Infected Patients (%)	Number of Uninfected Patients
**None**	61 (48.4)	1925 (68)
**Chloroquine**	5 (4)	127 (4.5)
**Mefloquine**	2 (1.6)	135 (4.8)
**Doxycycline**	7 (5.6)	100 (3.5)
**Malarone**	2 (1.6)	396 (14)
**More than 1 type of prophylaxis used**	3 (2.4)	-
**Other**	12 (9.5)	146 (5.2)
**N/A**	34 (27)	-

At the time of the specimen submission, 53.2% of patients were on malaria treatment. Different types of treatments regimens prescribed included the following drugs sometimes used in combination for severe cases: Doxycycline (n = 19), Quinine (n = 24), Atovaquone-Proguanil (n = 23), Chloroquine (n = 9), Artesunate (n = 4), Primaquine (n = 3), Clindamycin (n = 2), Mefloquine (n = 2). Fourteen patients indicated that they have received the treatment but did not list the name of the treatment, 2 of these patients reasoned that this was because they were not able to recall the name of the treatment received. Twelve patients indicated they have received the treatment but failed to indicate the name of the treatment

### Parasitemia and severe malaria

Based on one definition of severe *P. falciparum* malaria (parasitaemia level of >2%) as outlined in the WHO Guidelines for the Treatment of Malaria, there were 19 severe *P. falciparum* patients [20]. GMP of the severe malaria patients were 4.41%.The mean age of the severe malaria patients were 33.07±3.34 years, and 68.4% of the cases were males. Of the 19 cases, 15 patients have provided their travel history. All 15 patients listed African countries as their potential malaria exposure region, and 5 of them had visited Sudan. Of the 19 cases, 14 patients provided their clinical history. 8 patients took prophylaxis, including chloroquine (n = 2), artemether-lumefantrine (n = 1), atovaquone-proguanil (n = 1), chloroquine-proguanil (n = 1), and unknown (n = 3). The most common approach for treating the severe malaria cases was quinine-based combination therapy at the time of writing (n = 4), although this has now shifted to artesunate combination therapy. No deaths were noted in this study.

### Estimate of Malaria Incidence Rate

**Figure 4:**
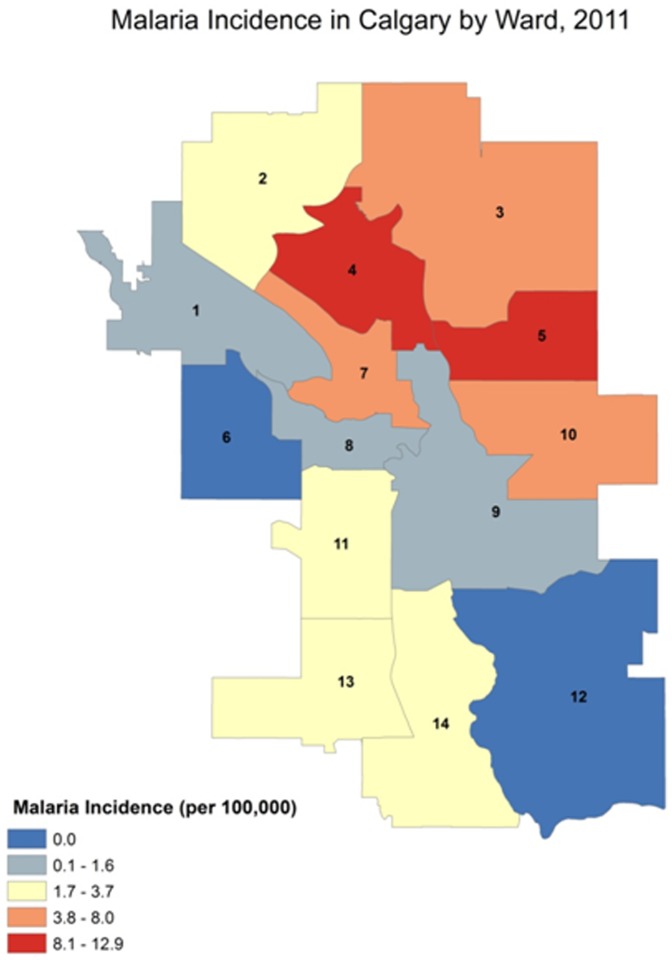
City of Calgary map indicating malaria incidence by ward based on 2011 census data.

The malaria incidence rates between the 14 wards defined by the City of the Calgary varied greatly (Figure 4). The highest incidence rate was observed in ward 5 (12.88/100,000), followed by ward 4 (8.28/100,000), 3(5.56/100,000), and 10 (5.42/100,000). Wards 3, 4, 5, and 10 are mainly clustered in the North East quadrant (Figure 4). The wards located in the North West, South East, and South West quadrants showed relatively low incidence rate of below 5/100,000, and no malaria cases were found in ward 6 and 12. The percentage of prophylaxis usage in the wards that had an incidence rate above 5/100,000 was 15.4%, compared to 50.0% in the wards that had incidence rate below 5/100,000. However, the percentage differences across the groups did not reach statistical significance (p = 0.12), this is most likely due to the small sample size and limited prophylaxis history information.

In order to determine the relationship between malaria incidence rates and the socioeconomic status, social districts which essentially overlap with wards (Figure 4) were evaluated (Table 4). The highest incidence rates were observed in the upper North East (9.20/100,000), followed by the North (7.98/100,000), and the lower North East (7.98/100,000) districts. The rest of the districts exhibited either incidence rates below 5/100,000, or no incidence of malaria. When the two groups (i.e. social districts that exhibited malaria incidence rate higher than 5/100,000, and those with lower than 5/100,000) were compared, certain socioeconomic disadvantages were associated with the higher malaria incidence rate. The average percentage of people with a university certificate/diploma/or degree was significantly lower in the top three highest malaria incidence rate social districts than in the low malaria incidence rate social districts (24.7% vs. 41.5%, p = 0.05). In addition, the average number of immigrants residing in the high malaria incidence rate social districts was significantly higher than in the low malaria incidence rate social districts (23,573 vs. 14,308, p = 0.02) and tended to come from malaria-endemic parts of Asia and Africa. Additionally, 3 of the 4 social districts with the lowest percent with university level education had the highest malaria incidence rates. In contrast, the difference between the average annual income in the two groups were statistically indistinguishable (81,077 vs. 110,233, p = 0.07). Overall, there has been a trend of increasing malaria incidence rate in Calgary. Retracing back to 2001, the malaria incidence rate was 2.40/100,000. Five years later in 2006, the rate has slightly increased to 2.82/100,000. Recently in 2011, the incidence rate was 4.31/100,000, which is a nearly a 2 fold increase over the study period.

**Table 4:** Malaria Incident Rates (2011) when compared Socioeconomic Data for Calgary Social Districts.

Social District	2011 Incidence Rate (per 100,000)	Percent with University certificate/Diploma/Degree	Average Income	Total Number of Immigrants	Top 5 Places of Birth for Recent immigrants	Ward Number
CALGARY - CENTRE WEST	0.00	44	$101,811.00	9896	E Asia, E Europe, SE Asia, S Asia, Eastern Africa	6,8,11
CALGARY - LOWER NW	0.00	55	$131,108.00	11098	E Asia, S Asia, S America, SE Asia, E Europe	1
CALGARY - SW	1.08	36	$110,220.00	17378	E Asia, SE Asia, E Europe, S Asia, N Europe	13,14
CALGARY - SE	1.29	32	$118,052.00	9274	N Europe, SE Asia, E Europe, E Asia, S Asia	9,12
CALGARY - WEST	1.29	53	$151,792.00	14840	E Asia, SE Asia, E Europe, South America, S Asia	1,6,8
CALGARY - CENTRE	1.81	48	$86,301.00	13706	E Asia, Eastern Africa, E Europe, WC Asia & Mid East, SE Asia	7,8,9
CALGARY - UPPER NW	1.96	49	$125,596.00	28389	E Asia, S Asia, WC Asia & Mid East, Europe, SE Asia	1,2,4,7
CALGARY - ELBOW	2.46	56	$136,799.00	7719	E Asia, E Europe, S Asia, SE Asia, South America	8,9,11
CALGARY - CENTRE NORTH	3.37	44	$102,498.00	9318	E Asia, S Asia, E Europe, N Africa, E Africa	1,7,9
CALGARY - FISH CREEK	3.42	34	$107,800.00	20102	E Asia, E Europe, SE Asia, WC Asia & Mid East, South America	9,11,13,14
CALGARY - NOSEHILL	3.95	35	$88,012.00	13662	E Asia, SE Asia, S America, N Europe, WC Asia & Mid East	4,7,9
CALGARY - EAST	4.08	12	$62,801.00	16311	SE Asia, N Africa, S Asia, E Asia, E Africa	9,10
CALGARY - LOWER NE	7.25	19	$73,330.00	28196	S Asia, SE Asia, WC Asia & Mid East, E Asia, Northern Africa	5,10
CALGARY - NORTH	7.98	33	$96,328.00	20201	E Asia, S Asia, SE Asia, WC Asia & Mid East, S America	2,3,4
CALGARY - UPPER NE	9.20	22	$73,572.00	22322	S Asia, SE Asia, WC Asia & Mid East, East Africa, East Asia	3,5

## Discussion

Human mobility is essential to the survival and dissemination of the malaria parasite [21]. Centralized regional testing of malaria presents a unique opportunity to accurately estimate the true burden of infectious diseases in a given population. The growing stature of the city of Calgary driven by the burgeoning oil sands industry has meant increased travel to and from Calgary and, in large part, this includes recent immigrants who visit friends and relatives (VFR) in areas of malaria endemicity. Our retrospective study catalogues an increasing trend of imported malaria that is similar to that reported in other major centres in Canada [6], [22]. Our testing modality which relies on both expert microscopy and PCR confirmation provides an accurate diagnostic database coupled to a patient travel questionnaire detailing, for example, country of travel and prophylactic drug taken.

Taken together, the data point to a troubling correlation where individuals in areas of the city with increased immigration, lower levels of education, and lower average income do not access the health care system for prophylaxis recommendations and thus acquire more malaria. For example, the North East sector of our city has the highest total number of immigrants based on census data and also the lowest average income. In contrast, the Northwest of our city has a significant number of immigrants but higher levels of income and we assume education levels. Census data conducted by health authorities in this province of Canada, also point to increased immigration from South Asia and Africa in the north east of city where the highest cases of malaria occur. This observation has been made before but not quantified in terms of incident rates and correlated to socioeconomic data in Canada [6], [22]. The trend observed here has also been observed in a similar study conducted recently in Barcelona [23]. Interestingly, in a UK 20 year observational study, travel to Africa for VFR was the highest risk factor for acquiring malaria but tourists, the elderly, and acquisition in areas less prone to malaria acquisition were actually associated with increased mortality [24]. Thus, it appears that African VFR as seen in our group may result in malaria but not necessarily life-threatening illness. Nevertheless, severe malaria did occur in close to a quarter of *P. falciparum* cases which were fortunately not associated with mortality.

While the reasons for traveling without prophylaxis are likely multifactorial, we speculate that cultural attitudes toward malaria play a significant role. Gender differences may also play a part as significantly more men acquire malaria then women, again perhaps due to adherence rates with prophylaxis as shown by others [25]. In fact, this conclusion was supported by a study looking into South Asian travelers returning “home” from the United Kingdom [13]. The majority individuals who tested positive for malaria were taking no prophylaxis. A very small minority were taking the first line drug recommended for malaria prophylaxis namely malarone [26]. Whether the costs associated with visiting a travel clinic for immunization, advice, and prescription drugs are a factor has not been clearly documented in this population. Clearly, visiting friends and relatives and new immigration were the single dominant epidemiological factor associated with malaria acquisition in Calgary. Previous work by our group has suggested immune responses differ in tourists who are malaria naive as compared to recent immigrant VFR [27]. In Africa, for example, it is not unusual to access a pharmacy for antimalarials without a prescription when fever presents. However, counterfeit drugs and inappropriate regimens are commonplace [28]. In our population, imported *P. falciparum* malaria is attributed to travel to West Africa and a higher geometric mean parasitemia, whereas *P. vivax* is linked to travel to South Asia with lower parasitemia. Surprisingly, the 25–29 age group were the single largest contributing age group for positive malaria cases, as opposed to infant or the elderly, and may be associated with patterns of non-adherence to prophylaxis regimens. Limitations of this study include the fact we do not have denominator data for rates of travel to regions to provide risk information. We also cannot provide data on rates of travel by districts in the city but assume higher rates of immigration correlate with increased travel given the fact that imported malaria is linked to VFR in our population. Given the localized nature of increased imported malaria incidence, a case can be made for more concerted public health intervention in these wards of the city through social media, physician offices, and cultural centers targeting both West African and South Asian immigrants.
